# Prolonged post-androgen abuse hypogonadism: potential mechanisms and a proposed standardized diagnosis

**DOI:** 10.3389/fendo.2025.1621558

**Published:** 2025-07-03

**Authors:** Joël van Os, Diederik Laurens Smit, Peter Bond, Willem de Ronde

**Affiliations:** ^1^ Spaarne Gasthuis, Department of Internal Medicine, Haarlem, Netherlands; ^2^ Android Health Clinic, Department of Performance and Image-enhancing Drugs Research, Utrecht, Netherlands

**Keywords:** androgen abuse, hypogonadism, anabolic steroid abuse, HPGA recovery, anabolic-androgenic steroids, testosterone (androgen), endocrine disruption

## Abstract

Androgen abuse, which is increasingly prevalent, inevitably leads to suppression of the hypothalamic-pituitary-gonadal axis (HPGA). While most individuals recover HPGA function following androgen cessation, a subset experiences prolonged hypogonadism, with symptoms persisting for months or even years. Currently, this condition lacks a standardized definition, complicating both diagnosis and treatment. In this article, we explore the potential mechanisms underlying prolonged hypogonadism after androgen abuse, including the role of prolonged androgen activity, hypothalamic-pituitary alterations, testicular changes, suppression of sex hormone-binding globulin (SHBG), genetic predisposition, and undisclosed ongoing androgen abuse. We propose the term ‘Prolonged Post-Androgen Abuse Hypogonadism’(PPAAH) to standardize diagnosis and guide future research. PPAAH is provisionally defined as persistent hypogonadism six months after cessation of androgen abuse, in individuals with a cumulative androgen exposure of at least 150 mg per week for a minimum of six months. Diagnosing PPAAH requires excluding other causes of hypogonadism. This preliminary framework is intended to support further research into the pathophysiology and management of this condition, and may require refinement as further evidence emerges.

## Introduction

Androgen abuse is relatively common, with an estimated prevalence of up to 6% and indications of a rising trend (1–3). Androgens, also referred to as anabolic-androgenic steroids, encompass a group of compounds structurally similar to testosterone that exert similar physiological effects. These substances are mainly abused for their ability to significantly enhance muscle mass and strength, especially when combined with strength training (4). The typical androgen abuser is male, aged 20 to 40 years, and engaged in bodybuilding or weightlifting (5).

Androgens are abused in varying patterns and dosages, often far exceeding physiological levels. Commonly, users follow a ‘cycle’, typically lasting from 6 to 18 weeks, after which androgen abuse is (temporarily) halted. A growing number of users adopt a ‘blast and cruise’ approach, alternating between high-dose ‘blasts’ and lower-dose ‘cruises’, continuing androgen abuse without full cessation. This strategy aims to maintain muscle mass between cycles and avoid symptoms of testosterone deficiency (5, 6). Most cycles contain an injectable testosterone-ester combined with one or more other compounds, most commonly nandrolone, trenbolone, drostanolone and/or boldenone esters. Without esterifications, androgens rapidly enter the bloodstream after administration, resulting in high peak levels and a very short plasma half-life. To improve pharmacokinetics, a carboxylic acid is attached to the steroid. The greater the lipophilicity of the carboxylic acid, the slower the release from the injected depot. The most commonly used esters are propionate, enanthate, undecanoate, undecylenate, and acetate (7).

During androgen abuse, there is an inevitable impairment of testosterone production and spermatogenesis due to suppression of the hypothalamic-pituitary-gonadal axis (HPGA). This suppression can be explained by excessive circulating androgens exerting negative feedback at the hypothalamic level (8). Additionally, certain compounds exert progestogenic or estrogenic effects, either directly or through enzymatic conversion into bioactive metabolites. Both progestogen and estrogen are known to have strong suppressive effects on the male HPGA (9–11).

Upon discontinuation of androgens, large interindividual variability in the duration until HPGA recovery has been reported. While some recover within weeks, recovery can remain incomplete in others after years of cessation (12–14). This variability is to be expected, given differences in the half-lives of the androgens abused and the fact that HPGA recovery only begins once circulating androgen levels fall below a certain threshold. However, incomplete recovery lasting a year or more cannot be fully explained by these factors. Although higher cumulative lifetime androgen exposure increases the likelihood of impaired recovery, the exact mechanisms underlying this phenomenon remain poorly understood (15). Former androgen abusers often complain of symptoms indicative of hypogonadism and may seek medical attention. In fact, a study investigating hypogonadal men at an academic-based urology practice found that androgen-induced hypogonadism was the most common etiology of profound hypogonadism (defined as total testosterone at or below 50 ng/dL or 1.73 nmol/L) (16). Despite its prevalence, androgen-induced hypogonadism is an underrecognized issue and although impeded HPGA recovery following androgen abuse has been described in the literature, there is no clear consensus on the diagnosis or management (12, 14, 17–24). A standardized syndrome definition is necessary to provide a foundation for further research. Given the suggested rise in androgen abuse, understanding the pathophysiology of androgen-induced hypogonadism is critical for improving clinical care for this growing patient population.

In this article, we begin by exploring seven potential mechanisms involved in prolonged hypogonadism following androgen cessation, which we categorize into four hypotheses and three unlikely explanations. The latter are deemed insufficient to explain the hypogonadal symptoms reported by former androgen abusers. An overview of these mechanisms is presented in **Table 1**. Subsequently, we propose a universal definition for a syndrome termed ‘prolonged post-androgen abuse hypogonadism’ (PPAAH) to guide future research and clinical practice.

**Table 1:** Potential mechanisms involved in prolonged hypogonadism following androgen abuse.

Hypotheses	Unlikely explanations
Changes at the pituitary and/or hypothalamic levels (14, 25, 26)	Prolonged activity of exogenous androgens or their metabolites (27, 28)
Testicular changes (29, 30)	Reduced SHBG levels (31)
Changes at multiple HPGA levels (22)	Undisclosed continued androgen abuse
Genetic predisposition to HPGA disturbances (32)	

## Approach to literature selection

Relevant studies were identified through a non-systemic literature search in PubMed, focusing on human studies published in English. Selection criteria were based on the relevance to androgen abuse and its endocrine consequences. The following keywords and MeSH terms were used in various combinations: “anabolic steroids”, “anabolic androgenic steroids”, “androgen abuse”, “androgen-induced hypogonadism”, and “Hypothalamic-Pituitary-Gonadal Axis”.

Additional references were identified by manually screening the reference lists of included articles, with a particular focus on studies examining the pathophysiology, clinical manifestations, diagnostic strategies, and recovery trajectories of hypogonadism following androgen cessation.

## Hypotheses for prolonged hypogonadism

### Hypothesis 1: changes at the pituitary and/or hypothalamic levels

Hypogonadism following androgen abuse is typically characterized by hypogonadotropic hypogonadism, suggesting androgen-induced alterations to the pituitary and/or hypothalamus (17). A case study by Jarow et al. (1990) documented two patients with hypogonadotropic hypogonadism more than a year after androgen cessation. Both patients demonstrated a blunted pituitary response to a bolus of gonadotropin-releasing hormone (GnRH) compared to a healthy male (14). Additionally, other case studies have demonstrated positive effects of either GnRH or GnRH agonists on HPGA recovery (25, 26). While these findings support the hypothesis of androgen-induced pituitary or hypothalamic changes, they do not clarify whether the primary dysfunction occurs at the pituitary, hypothalamic, or both levels, nor do they elucidate the precise mechanisms involved. Furthermore, other potential causes of hypogonadotropic hypogonadism were not fully excluded in these studies.

One potential mechanism involves alterations in the kisspeptin-neurokinin B-dynorphin (KNDy) network. Discovered a few decades ago, this network of neuropeptides and neurons is now recognized as crucial for HPGA function (33, 34). KNDy neurons project directly to GnRH neurons, and increasing evidence suggests that kisspeptin and neurokinin B are essential for normal GnRH secretion. Research has demonstrated that KNDy neurons, unlike GnRH neurons, express androgen, estrogen and progesterone receptors and that steroid-induced negative feedback affects kisspeptin receptor (KISS1R) expression. Inactivation of this receptor has been shown to result in hypogonadotropic hypogonadism, the hormonal state also observed after androgen cessation (33–38). Nevertheless, the precise role of the KNDy network, particularly in males, remains incompletely understood, but future research into this network may contribute to a better understanding of HPGA recovery (39).

Evidence opposing this hypothesis comes from a recent cross-sectional study by Bulut et al. (2025) (29). In this study, former androgen abusers (on average two years post-cessation) were compared with healthy controls. While total testosterone levels and sexual function were significantly lower in former abusers, no differences were observed in pituitary responses to GnRH stimulation. The authors primarily found indication of impaired testicular function, rather than central HPGA dysfunction.

### Hypothesis 2: testicular changes

Androgen abuse is known to induce changes to testicular structure and function (40). The suppression of luteinizing hormone (LH) and follicle-stimulating hormone (FSH) during androgen abuse disrupts normal testicular physiology. Reduced FSH impairs stimulation of Sertoli cells, which are essential for spermatogenesis. Simultaneously, low LH levels lead to a marked reduction in intratesticular testosterone concentrations – typically around 100 times higher than circulating levels (41, 42). This drop in local testosterone further compromises Sertoli cell function and impairs spermatogenesis, even when serum testosterone levels appear normal or supraphysiological.

These intratesticular hormonal changes are often accompanied by a reduction in testicular size, which frequently persists long after androgen cessation (40). The sustained reduction in testicular size might indicate irreversible damage to the testes. Alternatively, LH and FSH levels post-androgen cessation may be insufficient to restore testicular size to its pre-abuse state.

In studies on individuals with gender dysphoria undergoing male-to-female transition, cross-sex hormone therapy, often consisting of supraphysiological estradiol levels, results in HPGA suppression and testicular changes similar to those observed in androgen abusers. Histopathological evaluations of orchiectomy specimens from these patients, mostly years after the start of hormone treatment, reveal significant alterations, including hypoplastic or absent Leydig cells (43–47).

To investigate the testicular changes in androgen abusers, a study conducted by Rasmussen et al. (2021) measured insulin-like factor 3 (INSL3) levels, a biomarker indicative of Leydig cell function, in current and former androgen abusers compared to controls. INSL3 levels were significantly decreased in formers users, even years after cessation, with a negative correlation between androgen abuse duration and INSL3 levels, indicating persistent Leydig cell impairment (30). In a subsequent study by the same research group, Bulut et al. (2025) found that former androgen abusers exhibited impaired erectile function, a blunted testicular response to human chorionic gonadotropin (hCG) stimulation, and persistently decreased INSL3 levels, suggesting sustained impairment of Leydig cell capacity approximately two years post-cessation (29).

However, testicular damage alone is unlikely to fully explain HPGA dysfunction, as testicular failure typically presents with *hyper*gonadotropic hypogonadism (elevated LH and FSH), contrasting with the *hypo*gonadotropic hypogonadism seen post-androgen cessation (48). Thus, the testicular changes reported may therefore be secondary to central (hypothalamic or pituitary) alterations induced by androgens. Notably, the former androgen abusers in the study by Bulut et al. (2025) were not hypogonadal (mean testosterone ~14 nmol/L), suggesting that although prolonged impairment of testicular function is evident, this alone may be insufficient to cause hypogonadism (29).

### Hypotheses 3: changes at multiple HPGA levels

Previously, we discussed hypotheses regarding changes at hypothalamic-pituitary or testicular levels independently, leading to the hypothesis that androgen abuse induces changes at both of these levels. A study by Flanagan et al. (2015) provides support for this hypothesis. The study demonstrated impaired stimulation responses to both GnRH and hCG, a direct stimulator of testicular testosterone production, in a group of 13 long-term androgen abusers with persistent hypogonadism at least seven months post-cessation (22). The authors noted variability in responses, suggesting that androgens may impair HPGA recovery through multiple pathways, further complicating its understanding.

A different study by Martikainen et al. (1985), examined testicular responsiveness to a single dose of hCG in six healthy adult men three weeks after androgen cessation, during which they exhibited (transient) hypogonadotropic hypogonadism (49). These men demonstrated blunted testosterone responses to hCG, and the responses were similar to those observed in prepubertal boys and adult men with isolated gonadotropin deficiency (50, 51). In eugonadal adult men, the testosterone response to hCG typically follows a biphasic pattern, with a modest increase occurring 2–4 hours post-injection and a more substantial rise after 72–96 hours, the latter being preceded by peaks in serum estradiol and the testosterone precursor 17-hydroxyprogesterone. This pattern is thought to be related to estradiol-mediated inhibition of 17,20-desmolase activity (52). However, the men in the study exhibited no increases in 17-hydroxyprogesterone or estradiol, which may be attributable to a diminished estradiol-synthesizing capacity of the Leydig cells due to prolonged gonadotropin deprivation during androgen abuse.

### Hypothesis 4: genetic predisposition to HPGA disturbances

The fact that most individuals achieve full HPGA recovery after androgen abuse while others do not, raises the possibility of genetic susceptibility to HPGA dysfunction in the non-recovering individuals. Caronia et al. (2011) found heterozygous mutations associated with hypogonadotropic hypogonadism in female individuals with functional hypothalamic amenorrhea (32). It is conceivable that a similar genetic predisposition may impair HPGA recovery in former androgen abusers, although no studies have yet examined this in detail.

## Unlikely explanations for prolonged hypogonadism

After discussing various hypotheses for prolonged hypogonadism following androgen abuse, we now explore different mechanisms involved in HPGA recovery post-androgen cessation. However, these mechanisms fail to fully account for the persistent symptoms of hypogonadism reported by former abusers, and are thus considered ‘unlikely explanations’.

### Unlikely explanation 1: Prolonged activity of exogenous androgens or their metabolites

Given that androgens suppress the HPGA, one could hypothesize that hypogonadism following androgen abuse results from the prolonged activity of exogenous androgens or their metabolites, rather than androgen-induced dysfunction of the HPGA itself. Theoretically, HPGA recovery should commence once circulating androgenic, estrogenic and/or progestogenic levels fall below a certain threshold. The rate at which this occurs would depend on the half-life of the abused androgen. However, the quality of the abused androgens varies considerably, with significant disparities between the labelled and actual content, including differences in compound type and dosage (7). Consequently, users may inadvertently administer an androgen with a longer half-life than anticipated, potentially spanning multiple weeks.

However, even a half-life of several weeks does not account for the prolonged duration of HPGA recovery, which can extend close to a year or more in some individuals. Furthermore, the expected half-life of the administered androgen has not been shown to reliably predict recovery duration in a group of androgen abusers, mostly using a mix of illegally produced androgens in highly supraphysiological doses (13).

Notably, studies investigating 19-nortestosterone (nandrolone) hexyloxyphenylpropionate in healthy male volunteers have reported its presence or that of its metabolites in serum or urine for up to a year following cessation (27, 28). If these substances retain androgenic activity or continue to exert negative feedback effects on the HPGA, they could potentially explain the prolonged hypogonadotropic hypogonadism observed in some former androgen abusers. However, the persistence of androgenic activity does not explain the hypogonadal symptoms reported by former abusers. Moreover, a study by Flanagan et al. (2015), which specifically investigated the presence of androgens, including nandrolone and its metabolites, in a group of hypogonadal former androgen abusers at least 7 months post-cessation, found no evidence of their presence (22). Thus, this explanation is not able to explain all cases of hypogonadism following androgen abuse.

The possibility that estrogen contributes to prolonged HPGA suppression forms the basis for the widespread use of ‘post-cycle therapy’ (PCT) by androgen abusers, intended to accelerate HPGA recovery and prevent symptoms of testosterone deficiency. PCT typically consists of an aromatase inhibitor (AI), a selective estrogen receptor modulator (SERM) and/or human chorionic gonadotropin (hCG), used for several weeks. However, despite its widespread use, the effectiveness of PCT in facilitating HPGA recovery remains unproven (7, 13).

### Unlikely explanation 2: Reduced levels of sex hormone-binding globulin

Approximately 2% of testosterone in plasma is unbound (‘free’) and is hypothesized to actively exert physiological effects. Testosterone is mostly bound to carrier proteins, with SHBG being the main carrier protein for circulating testosterone. A reduction in SHBG levels leads to a decrease in total testosterone, which could unjustly suggest hypogonadism, even if free testosterone levels remain normal. This phenomenon, termed pseudo-hypogonadism by some, is observed in association with conditions like obesity and does not necessarily imply true androgen deficiency (53).

Androgen administration is known to suppress SHBG, and this suppression may persist many months after cessation. A study by Handelsman et al. (2022) investigated the recovery of male reproductive endocrine function in 303 men with glucose intolerance but no pathological hypogonadism, who completed a 2-year placebo-controlled clinical trial of testosterone undecanoate. The study found that, from 24 weeks after last testosterone injection, total testosterone levels in the testosterone-treated group were significantly lower than in the placebo group. This difference persisted throughout the follow-up period, which extended to 64 weeks post-injection. Notably, SHBG levels remained significantly lower in the testosterone-treated group both during treatment and throughout follow-up and after adjusting for the mean serum SHBG, the testosterone levels between groups were no longer significantly different (31).

Consequently, low total testosterone levels observed in former androgen abusers may represent a numerical artifact due to suppressed SHBG. However, this does not fully explain the persistence of clinical symptoms in these individuals.

### Unlikely explanation 3: Undisclosed continued androgen abuse

It is essential to consider undisclosed ongoing androgen abuse in cases of impaired HPGA recovery. Unfortunately, this is hard to exclude because comprehensive doping tests are expensive and rarely available in clinical practice. In the study by Flanagan et al. (2015), undisclosed androgen abuse was excluded by comprehensive laboratory and urinary testing in individuals with impaired HPGA recovery following androgen abuse (22). While this explanation may apply in selected cases, it is unlikely to account for impaired recovery in all individuals.

## Prolonged post-androgen abuse hypogonadism

In conclusion, the etiology of impaired HPGA recovery following androgen abuse is complex, potentially multifactorial, and remains incompletely understood. To better understand HPGA recovery, further research is imperative. The current literature on this topic is limited and employs varying definitions of normal and abnormal HPGA recovery following androgen abuse (16–18, 21, 23, 54). To facilitate consistent terminology in clinical and research contexts, we propose a provisional framework for defining impaired HPGA recovery following androgen abuse, which we term ‘prolonged post-androgen abuse hypogonadism’ (PPAAH).

We define PPAAH as a diagnosis of exclusion in patients who have cumulatively administered supraphysiological doses of androgens (cumulative dose ≥ 150 mg/week) for a minimum duration of six months, and who exhibit persistent hypogonadism at least six months after androgen cessation (see **Box 1**). Hypogonadism is defined as the presence of at least one symptom indicative of hypogonadism, combined with fasting morning (7–11 am) serum free testosterone levels below the lower limit of normal on two separate days. Common symptoms of hypogonadism include reduced sexual desire, erectile dysfunction, fatigue, sleep disturbance, depressed mood and reduced physical performance (55).

Box 1 **Diagnostic criteria for PPAAH.**

A diagnosis of PPAAH is established when **all** of the following criteria are met:

1. History of cumulative supraphysiological androgen abuse defined as ≥ 150 mg per week for a total duration of ≥ 6 months (not necessarily consecutive)

2. Persistent hypogonadism ≥ 6 months after androgen cessation, as evidenced by:

 • Fasting morning serum free testosterone levels below the lower limit of normal on two separate days

 • At least one symptom indicative of hypogonadism, such as: reduced sexual desire, erectile dysfunction, fatigue, sleep disturbance, depressed mood or reduced physical performance

3. Exclusion of other causes of hypogonadism, based on:

 • Laboratory evaluation: including serum prolactin, morning cortisol, IGF-1, TSH, free T4, serum iron, total iron-binding capacity (TIBC), transferrin saturation, and ferritin

 • Pituitary imaging: MRI of hypothalamic-pituitary region to rule out structural abnormalities

*The criteria listed here are provisional and intended to guide clinical and research discussion.*

*They do not yet constitute a formal or universally accepted diagnostic standard.*

### Rationale for diagnostic cut-offs

Given the paucity of robust data, the proposed thresholds should be regarded as preliminary and pragmatically chosen rather than empirically validated. They are intended to offer a workable foundation for clinical and research use and may require revision as more evidence becomes available.

Medically prescribed testosterone replacement therapy (TRT) typically involves weekly doses of 75 to 100 mg of testosterone (56). However, the androgens used by recreational abusers – often acquired through local dealers of the internet – are frequently of poor quality, possibly resulting in inconsistent dosing. To account for this variability, we define a weekly dose of ≥ 150 mg as supraphysiological (7).

We consider a cumulative duration of at least six months of supraphysiological androgen abuse to be the minimum threshold at which hypogonadism can reasonably be attributed to androgen exposure. This criterion remains open to debate and may be refined as more data emerge. Notably, both clinical experience and previous studies suggest that individuals presenting with prolonged hypogonadism typically report androgen abuse exceeding this six-month threshold (13).

The requirement of hypogonadism persisting for at least six months after androgen cessation was selected based on the assumption that, by this time, exogenous androgens are unlikely to remain in circulation at levels capable of exerting continued suppression of the HPGA. Moreover, prospective studies indicate that serum testosterone levels typically normalize within several months after cessation in individuals without underlying dysfunction (13, 57–63). Therefore, persistent hypogonadism at six months post-cessation is considered indicative of an underlying dysfunction of the HPGA.

### Free testosterone measurement

Serum free testosterone is considered a more accurate marker for diagnosing PPAAH rather than total serum testosterone due to the persistently reduced SHBG levels seen after androgen cessation.

When measuring free testosterone, several factors must be considered. The gold standard is liquid chromatography with tandem mass spectrometry (LC-MS/MS) coupled equilibrium dialysis. However, this technique is labour intensive, technically challenging, and not routinely available.

As a practical alternative, free testosterone can be calculated using total testosterone, SHBG, and albumin levels. Among the available formulas, the Vermeulen equation currently offers the most robust approximation. Nevertheless, several limitations warrant attention. First, the accuracy of the calculated free testosterone is dependent on the quality of the assays used to measure total testosterone and SHBG. Even with reliable assays, the Vermeulen method tends to slightly overestimate free testosterone levels (64).

Moreover, the calculation assumes a fixed binding affinity between SHBG and testosterone. Yet, SHBG polymorphisms have been shown to alter this binding affinity, potentially leading to discrepancies between calculated and true free testosterone levels (65). In addition, the presence of residual exogenous androgens, which also bind to SHBG and may contribute substantially to the total testosterone pool, can render the calculation unreliable (66). A cessation period of six months is theoretically sufficient to ensure that exogenous androgens are no longer circulating in significant amounts, except in cases of undisclosed androgen abuse.

Direct measurement of free testosterone via immunoassays is discouraged due to poor specificity and accuracy. These assays often correlate more strongly with total testosterone than with true free testosterone levels. Comparative studies have demonstrated weak agreement with the gold standard, prompting the Endocrine Society to recommend against their use in clinical settings (64, 67).

Finally, while no universal consensus exists on the cut-off value for low versus normal free testosterone, a threshold of 225 pmol/L (65 pg/mL) is commonly used (68).

### Exclusion of other causes

By defining PPAAH as a diagnosis of exclusion, other causes of hypogonadism should be ruled out.

Hypogonadism following androgen cessation typically presents as *hypo*gonadotropic hypogonadism, so especially in the case of *hyper*gonadotropic hypogonadism, another diagnosis should be considered. It is essential to check for pre-androgen administration testosterone levels, but if these are unavailable, each patient should be evaluated for signs of pre-existing hypogonadism, such as delayed puberty, maldescended testes, involuntary childlessness, anosmia, and gynaecomastia. Physical examination should focus on cryptorchidism, micropenis and a testicular volume below 8 ml, which is uncommon during or after androgen abuse and may indicate undiagnosed Klinefelter syndrome (21).

Additionally, laboratory evaluations should include testing for hyperprolactinemia, haemochromatosis, other markers of pituitary dysfunction, as well as magnetic resonance imaging (MRI) of the pituitary to exclude other causes of hypogonadotropic hypogonadism. Medication and drug use should be reviewed for substances known to affect gonadal function and clinicians should attempt to rule out undisclosed continued androgen abuse through doping tests if available. If such tests are not feasible, continued androgen abuse cannot be definitely excluded, and clinicians must remain vigilant for this possibility. While this uncertainty remains, previous research suggests that a negative self-reported history of androgen abuse – particularly when combined with serum LH measurement – offers relatively high diagnostic reliability. In this context, a suppressed LH level, especially in combination with persistently low serum high-density lipoprotein (HDL), low SHBG, and elevated hematocrit six months after supposed androgen cessation, should raise suspicion of ongoing abuse. However, these findings do not serve as conclusive proof (69, 70).

### Directions for future research

To advance our understanding of the pathophysiology underlying PPAAH, well-designed prospective studies are essential. An appropriate starting point would be longitudinal cohort studies in which individuals presenting with hypogonadism following androgen cessation are systematically monitored over time. These studies should aim to characterize the trajectory of HPGA recovery and to identify factors that predict either full recovery or persistent dysfunction.

Comprehensive baseline assessment should ideally include detailed hormonal profiling, pituitary imaging, and objective confirmation of androgen cessation through doping tests. In this context, a World Anti-Doping Agency (WADA)-accredited urine analysis, performed in a certified anti-doping laboratory, is considered the gold standard for the detection of androgen abuse and should be implemented where feasible.

In addition to conventional GnRH and hCG stimulation tests, emerging techniques such as kisspeptin stimulation or analyses of diurnal gonadotropin secretion patterns may offer deeper insights into the functional status of the hypothalamus and pituitary. Genetic testing may also be considered to explore potential hereditary predispositions to HPGA disturbances.

Longitudinal monitoring would allow for the identification of hormonal recovery patterns and could help uncover clinically meaningful predictors, such as duration and intensity of androgen abuse, age at onset, and the presence of pre-existing dysfunction. These studies would also present opportunities to evaluate the clinical utility of novel diagnostic tools and to assess potential interventions aimed at promoting HPGA recovery. Such efforts are essential to deepen our understanding of the mechanisms underlying prolonged hypogonadism after androgen cessation and to improve clinical outcomes for affected individuals.

## Conclusion

In summary, androgen abuse is associated with potentially long-lasting disruptions of the HPGA, presenting as hypogonadism in some individuals. HPGA recovery following androgen abuse is complex and not well understood, and its understanding is further complicated by the absence of a standardized definition distinguishing normal from abnormal recovery. Therefore, we propose the term ‘prolonged post-androgen abuse hypogonadism’ (PPAAH) as a provisional framework to support diagnostic consistency and facilitate future research and clinical management.

## Data Availability

The original contributions presented in the study are included in the article/supplementary material. Further inquiries can be directed to the corresponding author.

## References

[1] Sagoe D Molde H Andreassen CS Torsheim T Pallesen S. The global epidemiology of anabolic-androgenic steroid use: a meta-analysis and meta-regression analysis. Ann Epidemiol. (2014) 24:383–98. doi: 10.1016/J.ANNEPIDEM.2014.01.009 24582699

[2] Al Hashimi M Farahat Y Kandil H Al Khalidi I. Androgenic-anabolic steroid abuse trend and management: A prospective, cross-sectional, questionnaire-based survey. Health Sci Rep. (2023) 6(1):e1032. doi: 10.1002/HSR2.1032 36628108 PMC9827233

[3] Anawalt BD. Diagnosis and management of anabolic androgenic steroid use. J Clin Endocrinol Metab. (2019) 104:2490. doi: 10.1210/JC.2018-01882 30753550 PMC6517163

[4] Bhasin S Storer TW Berman N Callegari C Clevenger B Phillips J. The effects of supraphysiologic doses of testosterone on muscle size and strength in normal men. New Engl J Med. (1996) 335:1–7. doi: 10.1056/NEJM199607043350101/ASSET/C12E6A75-37D3-40BF-ADBF-D68237145055/ASSETS/IMAGES/LARGE/NEJM199607043350101_F1.JPG 8637535

[5] de Ronde W Smit DL. Anabolic androgenic steroid abuse in young males. Endocr Connect. (2020) 9:R102. doi: 10.1530/EC-19-0557 32229704 PMC7219134

[6] Idger De Zeeuw T Brunt M Van Amsterdam J Van De Ven K Van Den Brink W. Anabolic androgenic steroid use patterns and steroid use disorders in a sample of male gym visitors. European Addiction Research (2023) 29(2):99–108. doi: 10.1159/000528256 PMC10273855 36731448

[7] Smit DL de Hon O Venhuis BJ den Heijer M de Ronde W. Baseline characteristics of the HAARLEM study: 100 male amateur athletes using anabolic androgenic steroids. Scand J Med Sci Sports. (2020) 30:531–9. doi: 10.1111/SMS.13592 31663164

[8] Pitteloud N Dwyer AA Decruz S Lee H Boepple PA Crowley WF Jr. Inhibition of luteinizing hormone secretion by testosterone in men requires aromatization for its pituitary but not its hypothalamic effects: evidence from the tandem study of normal and gonadotropin-releasing hormone-deficient men. J Clin Endocrinol Metab. (2008) 93:784. doi: 10.1210/JC.2007-2156 18073301 PMC2266963

[9] Raven G De Jong FH Kaufman JM De Ronde W. In men, peripheral estradiol levels directly reflect the action of estrogens at the hypothalamo-pituitary level to inhibit gonadotropin secretion. J Clin Endocrinol Metab. (2006) 91:3324–8. doi: 10.1210/JC.2006-0462 16787981

[10] Finkelstein JS O’Dea LSL Whitcomb RW Crowley WF. Sex steroid control of gonadotropin secretion in the human male. II. Effects of estradiol administration in normal and gonadotropin-releasing hormone-deficient men. J Clin Endocrinol Metab. (1991) 73:621–8. doi: 10.1210/JCEM-73-3-621 1908485

[11] Brady BM Anderson RA Kinniburgh D Baird DT. Demonstration of progesterone receptor-mediated gonadotrophin suppression in the human male. Clin Endocrinol (Oxf). (2003) 58(4):506–12. doi: 10.1046/J.1365-2265.2003.01751.X 12641635

[12] Solanki P Eu B Smith J Allan C Lee K. Physical, psychological and biochemical recovery from anabolic steroid-induced hypogonadism: a scoping review. Endocr Connect. (2023) 12(12):e230358. doi: 10.1530/EC-23-0358 37855241 PMC10620455

[13] Smit DL Buijs MM De Hon O Den Heijer M De Ronde W. Disruption and recovery of testicular function during and after androgen abuse: the HAARLEM study. Hum Reprod. (2021) 36:880–90. doi: 10.1093/HUMREP/DEAA366 33550376

[14] Jarow JP Lipshultz LI. Anabolic steroid-induced hypogonadotropic hypogonadism. Am J Sports Med. (1990) 18:429–31. doi: 10.1177/036354659001800417 2403193

[15] Karila T Hovatta O Seppälä T. Concomitant abuse of anabolic androgenic steroids and human chorionic gonadotrophin impairs spermatogenesis in power athletes. Int J Sports Med. (2004) 25:257–63. doi: 10.1055/S-2004-819936 15162244

[16] Coward RM Rajanahally S Kovac JR Smith RP Pastuszak AW Lipshultz LI. Anabolic steroid induced hypogonadism in young men. J Urol. (2013) 190:2200–5. doi: 10.1016/J.JURO.2013.06.010 23764075

[17] Kanayama G Hudson JI DeLuca J Isaacs S Baggish A Weiner R. Prolonged hypogonadism in males following withdrawal from anabolic-androgenic steroids: an under-recognized problem. Addict (Abingdon England). (2015) 110:823–31. doi: 10.1111/ADD.12850 PMC4398624 25598171

[18] Rasmussen JJ Selmer C Østergren PB Pedersen KB Schou M Gustafsson F. Former abusers of anabolic androgenic steroids exhibit decreased testosterone levels and hypogonadal symptoms years after cessation: A case-control study. PloS One. (2016) 11(8):e0161208. doi: 10.1371/JOURNAL.PONE.0161208 27532478 PMC4988681

[19] Christou MA Christou PA Markozannes G Tsatsoulis A Mastorakos G Tigas S. Effects of anabolic androgenic steroids on the reproductive system of athletes and recreational users: A systematic review and meta-analysis. Sports Med. (2017) 47:1869–83. doi: 10.1007/S40279-017-0709-Z 28258581

[20] Bond P Smit DL de Ronde W. Anabolic–androgenic steroids: How do they work and what are the risks? Front Endocrinol (Lausanne). (2022) 13:1059473. doi: 10.3389/FENDO.2022.1059473 36644692 PMC9837614

[21] Botman E Smit DL de Ronde W. Clinical question: How to manage symptoms of hypogonadism in patients after androgen abuse? Clin Endocrinol (Oxf). (2023) 98:469–72. doi: 10.1111/CEN.14686 35133022

[22] Flanagan JN Lehtihet M. The response to gonadotropin-releasing hormone and hCG in men with prior chronic androgen steroid abuse and clinical hypogonadism. Horm Metab Res. (2015) 47:668–73. doi: 10.1055/S-0034-1398492 25642745

[23] Vilar Neto JO da Silva CA da Silva CAB Pinto DV de Sá Roriz Caminha J de Matos RS. Anabolic androgenic steroid-induced hypogonadism, a reversible condition in male individuals? A systematic review. Andrologia. (2021) 53(7):e14062. doi: 10.1111/AND.14062 33887077

[24] Grant B Campbell J Pradeep A Burns AD Bassett P Abbara A. Factors predicting normalization of reproductive hormones after cessation of anabolic-androgenic steroids in men: a single center retrospective study. Eur J Endocrinol. (2023) 189:601–10. doi: 10.1093/EJENDO/LVAD164 38102386

[25] Van Breda E Keizer HA Kuipers H Wolffenbuttel BHR. Androgenic anabolic steroid use and severe hypothalamic-pituitary dysfunction: a case study. Int J Sports Med. (2003) 24:195–6. doi: 10.1055/S-2003-39089 12740738

[26] Pirola I Cappelli C Delbarba A Scalvini T Agosti B Assanelli D. Anabolic steroids purchased on the Internet as a cause of prolonged hypogonadotropic hypogonadism. Fertil Steril. (2010) 94:2331.e1–2331.e3: doi: 10.1016/J.FERTNSTERT.2010.03.042 20416868

[27] Gårevik N Strahm E Garle M Lundmark J Ståhle L Ekström L. Long term perturbation of endocrine parameters and cholesterol metabolism after discontinued abuse of anabolic androgenic steroids. J Steroid Biochem Mol Biol. (2011) 127:295–300. doi: 10.1016/J.JSBMB.2011.08.005 21884791

[28] Knuth UA Behre H Belkien L Bents H Nieschlag E. Clinical trial of 19-nortestosterone-hexoxyphenylpropionate (Anadur) for male fertility regulation. Fertil Steril. (1985) 44:814–21. doi: 10.1016/S0015-0282(16)49043-6 3935486

[29] Bulut Y Brandt-Jacobsen N Madsen R Thevis M Frystyk J Albrethsen J. Characterization of leydig cell dysfunction in previous illicit androgen users. J Clin Endocrinol Metab. (2025) 00:1–10. doi: 10.1210/CLINEM/DGAF157 40052766

[30] Rasmussen JJ Albrethsen J Frandsen MN Jørgensen N Juul A Kistorp C. Serum insulin-like factor 3 levels are reduced in former androgen users, suggesting impaired leydig cell capacity. J Clin Endocrinol Metab. (2021) 106:E2664–72. doi: 10.1210/CLINEM/DGAB129 33693710

[31] Handelsman DJ Desai R Conway AJ Shankara-Narayana N Stuckey BGA Inder WJ. Recovery of male reproductive endocrine function after ceasing prolonged testosterone undecanoate injections. Eur J Endocrinol. (2022) 186:307–18. doi: 10.1530/EJE-21-0608 35000898

[32] Caronia LM Martin C Welt CK. A genetic basis for functional hypothalamic amenorrhea. N Engl J Med. (2011) 364:215–25. doi: 10.1056/NEJMOA0911064 PMC3045842 21247312

[33] Caronia LM Martin C Welt CK Sykiotis GP Quinton R Thambundit A. 60 YEARS OF NEUROENDOCRINOLOGY: The hypothalamo-pituitary-gonadal axis. J Endocrinol. (2015) 226:T41–54. doi: 10.1530/JOE-15-0113 PMC4498991 25901041

[34] Marques P Skorupskaite K Rozario KS Anderson RA George JT. Physiology of GnRH and gonadotropin secretion. In: Endotext. South Dartmouth, Massachusetts, USA: MDText.com, Inc. (2022). Available at: https://www.ncbi.nlm.nih.gov/books/NBK279070/.

[35] Smith JT Dungan HM Stoll EA Gottsch ML Braun RE Eacker SM. Differential regulation of KiSS-1 mRNA expression by sex steroids in the brain of the male mouse. Endocrinology. (2005) 146:2976–84. doi: 10.1210/EN.2005-0323 15831567

[36] Sharma A Thaventhiran T Minhas S Dhillo WS Jayasena CN. Kisspeptin and testicular function—Is it necessary? Int J Mol Sci. (2020) 21(8):2958. doi: 10.3390/IJMS21082958 32331420 PMC7216047

[37] Topaloglu AK Tello JA Kotan LD Ozbek MN Yilmaz MB Erdogan S. Inactivating KISS1 mutation and hypogonadotropic hypogonadism. N Engl J Med. (2012) 366:629–35. doi: 10.1056/NEJMOA1111184 22335740

[38] Lehman MN Coolen LM Goodman RL. Minireview: kisspeptin/neurokinin B/dynorphin (KNDy) cells of the arcuate nucleus: A central node in the control of gonadotropin-releasing hormone secretion. Endocrinology. (2010) 151:3479. doi: 10.1210/EN.2010-0022 20501670 PMC2940527

[39] Xie Q Kang Y Zhang C Xie Y Wang C Liu J. The role of kisspeptin in the control of the hypothalamic-pituitary-gonadal axis and reproduction. Front Endocrinol (Lausanne). (2022) 13:925206. doi: 10.3389/FENDO.2022.925206 35837314 PMC9273750

[40] de Ronde W Smit DL. Anabolic–androgenic steroid abuse and testicular function in men; recent insights. Curr Opin Pharmacol. (2022) 67:102318. doi: 10.1016/J.COPH.2022.102318 36410315

[41] Jarow JP Chen H Rosner W Trentacoste S Zirkin BR. Assessment of the androgen environment within the human testis: minimally invasive method to obtain intratesticular fluid. J Androl. (2001) 22:640–5. doi: 10.1002/J.1939-4640.2001.TB02224.X 11451361

[42] Morse HC Horike N Rowley MJ Heller CG. Testosterone concentrations in testes of normal men: effects of testosterone propionate administration. J Clin Endocrinol Metab. (1973) 37:882–6. doi: 10.1210/JCEM-37-6-882 4753430

[43] Matoso A Khandakar B Yuan S Wu T Wang LJ Lombardo KA. Spectrum of findings in orchiectomy specimens of persons undergoing gender confirmation surgery. Hum Pathol. (2018) 76:91–9. doi: 10.1016/J.HUMPATH.2018.03.007 29555572

[44] Cornejo KM Oliva E Crotty R Sadow PM Devins K Wintner A. Clinicopathologic features and proposed grossing protocol of orchiectomy specimens performed for gender affirmation surgery. Hum Pathol. (2022) 127:21. doi: 10.1016/J.HUMPATH.2022.05.017 35660072 PMC9489654

[45] Schulze C. Response of the human testis to long-term estrogen treatment: morphology of Sertoli cells, Leydig cells and spermatogonial stem cells. Cell Tissue Res. (1988) 251:31–43. doi: 10.1007/BF00215444 3342442

[46] Schneider F Neuhaus N Wistuba J Zitzmann M Heß J Mahler D. Testicular functions and clinical characterization of patients with gender dysphoria (GD) undergoing sex reassignment surgery (SRS). J Sex Med. (2015) 12:2190–200. doi: 10.1111/JSM.13022 26559385

[47] Sapino A Pagani A Godano A Bussolati G. Effects of estrogens on the testis of transsexuals: a pathological and immunocytochemical study. Virchows Arch A Pathol Anat Histopathol. (1987) 411:409–14. doi: 10.1007/BF00735221 3116755

[48] Dandona P Rosenberg MT. A practical guide to male hypogonadism in the primary care setting. Int J Clin Pract. (2010) 64:682. doi: 10.1111/J.1742-1241.2010.02355.X 20518947 PMC2948422

[49] Martikainen H Alén M Rahkila P Vihko R. Testicular responsiveness to human chorionic gonadotrophin during transient hypogonadotrophic hypogonadism induced by androgenic/anabolic steroids in power athletes. J Steroid Biochem. (1986) 25:109–12. doi: 10.1016/0022-4731(86)90288-8 3747510

[50] Smals AGH Pieters GFFM Kloppenborg PWC Lozekoot DC Benraad TJ. Lack of a biphasic steroid response to single human chorionic gonadotropin administration in patients with isolated gonadotropin deficiency. J Clin Endocrinol Metab. (1980) 50:879–81. doi: 10.1210/JCEM-50-5-879 6768759

[51] Tapanainen J Martikainen H Dunkel L Perheentupa J Vihko R. Steroidogenic response to a single injection of hCG in pre- and early pubertal cryptorchid boys. Clin Endocrinol (Oxf). (1983) 18:355–62. doi: 10.1111/J.1365-2265.1983.TB00579.X 6135519

[52] Martikainen H Huhtaniemi I Vihko R. Response of peripheral serum sex steroids and some of their precursors to a single injection of hCG in adult men. Clin Endocrinol (Oxf). (1980) 13:157–66. doi: 10.1111/J.1365-2265.1980.TB01037.X 7192190

[53] Handelsman DJ. Androgen misuse and abuse. Endocr Rev. (2021) 42:457–501. doi: 10.1210/ENDREV/BNAB001 33484556

[54] Rahnema CD Lipshultz LI Crosnoe LE Kovac JR Kim ED. Anabolic steroid–induced hypogonadism: diagnosis and treatment. Fertil Steril. (2014) 101:1271–9. doi: 10.1016/J.FERTNSTERT.2014.02.002 24636400

[55] Araujo AB Esche GR Kupelian V O'Donnell AB Travison TG Williams RE. Prevalence of symptomatic androgen deficiency in men. J Clin Endocrinol Metab. (2007) 92:4241–7. doi: 10.1210/JC.2007-1245 17698901

[56] Bhasin S Brito JP Cunningham GR Hayes FJ Hodis HN Matsumoto AM. Testosterone therapy in men with hypogonadism: an endocrine society clinical practice guideline. J Clin Endocrinol Metab. (2018) 103:1715–44. doi: 10.1210/JC.2018-00229 29562364

[57] Alen M Rahkila P Reinilä M Vihko R. Androgenic-anabolic steroid effects on serum thyroid, pituitary and steroid hormones in athletes. Am J Sports Med. (1987) 15:357–61. doi: 10.1177/036354658701500411 3661817

[58] Ruokonen A Alén M Bolton N Vihko R. Response of serum testosterone and its precursor steroids, SHBG and CBG to anabolic steroid and testosterone self-administration in man. J Steroid Biochem. (1985) 23:33–8. doi: 10.1016/0022-4731(85)90257-2 3160892

[59] Remes K Vuopio P Järvinen M Härkönen M Adlercreutz H. Effect of short-term treatment with an anabolic steroid (methandienone) and dehydroepiandrosterone sulphate on plasma hormones, red cell volume and 2,3-diphosphoglycerate in athletes. Scand J Clin Lab Invest. (1977) 37:577–86. doi: 10.3109/00365517709100649 145651

[60] Small M Beastall GH Semple CG Cowan RA Forbes CD. Alteration of hormone levels in normal males given the anabolic steroid stanozolol. Clin Endocrinol (Oxf). (1984) 21:49–55. doi: 10.1111/J.1365-2265.1984.TB00135.X 6430603

[61] Bijlsma JWJ Duursma SA Thijssen JHH Huber O. Influence of nandrolondecanoate on the pituitary-gonadal axis in males. Acta Endocrinol (Copenh). (1982) 101:108–12. doi: 10.1530/ACTA.0.1010108 6812344

[62] Gårevik N Börjesson A Choong E Ekström L Lehtihet M. Impact of single-dose nandrolone decanoate on gonadotropins, blood lipids and HMG CoA reductase in healthy men. Andrologia. (2016) 48:595–600. doi: 10.1111/AND.12488 26370185

[63] Alén M Reinilä M Vihko R. Response of serum hormones to androgen administration in power athletes. Med Sci Sports Exerc. (1985) 17:354–9. 2991700

[64] Narinx N David K Walravens J Vermeersch P Claessens F Fiers T. Role of sex hormone-binding globulin in the free hormone hypothesis and the relevance of free testosterone in androgen physiology. Cell Mol Life Sci. (2022) 79(11):543. doi: 10.1007/S00018-022-04562-1 36205798 PMC11803068

[65] Nyamaah JA Narinx N Antonio L Vanderschueren D. Use of calculated free testosterone in men: advantages and limitations. Curr Opin Endocrinol Diabetes Obes. (2024) 31(6):230–5. doi: 10.1097/MED.0000000000000891 39445719

[66] Vermeulen A Verdonck L Kaufman JM. A critical evaluation of simple methods for the estimation of free testosterone in serum. J Clin Endocrinol Metab. (1999) 84:3666–72. doi: 10.1210/JCEM.84.10.6079 10523012

[67] Rosner W Auchus RJ Azziz R Sluss PM Raff H. Utility, limitations, and pitfalls in measuring testosterone: an endocrine society position statement. J Clin Endocrinol Metab. (2007) 92:405–13. doi: 10.1210/JC.2006-1864 17090633

[68] Jasuja R Pencina KM Spencer DJ Peng L Privat F Dhillo W. Reference intervals for free testosterone in adult men measured using a standardized equilibrium dialysis procedure. Andrology. (2023) 11:125–33. doi: 10.1111/ANDR.13310 36251328

[69] Christou GA Christou MA Žiberna L Christou KA. Indirect clinical markers for the detection of anabolic steroid abuse beyond the conventional doping control in athletes. Eur J Sport Sci. (2019) 19:1276–86. doi: 10.1080/17461391.2019.1587522 30880613

[70] Shankara-Narayana N Brooker L Goebel C Speers N Handelsman DJ. Reliability of drug history to verify androgen abuse in men. J Clin Endocrinol Metab. (2022) 107:e3790–6. doi: 10.1210/CLINEM/DGAC348 PMC9387685 35661889

